# National Mental Health Survey of India, 2016 - Rationale, design and methods

**DOI:** 10.1371/journal.pone.0205096

**Published:** 2018-10-25

**Authors:** Banandur S. Pradeep, Gopalkrishna Gururaj, Mathew Varghese, Vivek Benegal, Girish N. Rao, Gautham M. Sukumar, Senthil Amudhan, Banavaram Arvind, Satish Girimaji, Thennarasu K., Marimuthu P., Kommu John Vijayasagar, Binukumar Bhaskarapillai, Jagadisha Thirthalli, Santosh Loganathan, Naveen Kumar, Paulomi Sudhir, Veena A. Sathyanarayana, Kangkan Pathak, Lokesh Kumar Singh, Ritambhara Y. Mehta, Daya Ram, Shibukumar T. M., Arun Kokane, Lenin Singh R. K., Chavan B. S., Pradeep Sharma, Ramasubramanian C., Dalal P. K., Pradeep Kumar Saha, Sonia Pereira Deuri, Anjan Kumar Giri, Abhay Bhaskar Kavishvar, Vinod K. Sinha, Jayakrishnan Thavody, Rajni Chatterji, Brogen Singh Akoijam, Subhash Das, Amita Kashyap, Sathish R. V., Selvi M., Singh S. K., Vivek Agarwal, Raghunath Misra

**Affiliations:** 1 Department of Epidemiology, Centre for Public Health, National Institute of Mental Health and Neuro Sciences (NIMHANS), Bengaluru, Karnataka, India; 2 Department of Psychiatry, National Institute of Mental Health and Neuro Sciences (NIMHANS), Bengaluru, Karnataka, India; 3 Department of Psychiatry, Centre for Addiction Medicine, National Institute of Mental Health and Neuro Sciences (NIMHANS), Bengaluru, Karnataka, India; 4 Department of Child and Adolescent Psychiatry, National Institute of Mental Health and Neuro Sciences (NIMHANS), Bengaluru, Karnataka, India; 5 Department of Biostatistics, National Institute of Mental Health and Neuro Sciences (NIMHANS), Bengaluru, Karnataka, India; 6 Department of Clinical Psychology, National Institute of Mental Health and Neuro Sciences (NIMHANS), Bengaluru, Karnataka, India; 7 Department of Psychiatry, LGB Regional Institute of Mental Health, Tezpur, Assam, India; 8 Department of Psychiatry, All India Institute of Medical Sciences, Raipur, Chhattisgarh, India; 9 Department of Psychiatry, Government Medical College, Surat, Gujarat, India; 10 Department of Psychiatry, Central Institute of Psychiatry, Ranchi, Jharkhand, India; 11 Department of Psychiatry, IMHANS, Kozhikode, Kerala, India; 12 Department of Community Medicine and Family Medicine, All India Institute of Medical Sciences, Bhopal, Madya Pradesh, India; 13 Department of Psychiatry, Regional Institute of Medical Sciences, Imphal, Manipur, India; 14 Department of Psychiatry, Government Medical College and Hospital, Chandigarh, India; 15 Department of Psychiatry, Sawai Man Singh Medical College, Jaipur, Rajasthan, India; 16 State Nodal Officer Mental Health Program Office Tamil Nadu, India; 17 Department of Psychiatry, King George’s Medical University, Lucknow, Uttar Pradesh, India; 18 Department of Psychiatry, Institute of Psychiatry, Kolkata, West Bengal, India; 19 Department of Psychiatric Social Work, LGB Regional Institute of Mental Health, Tezpur, Assam, India; 20 Department of Community Medicine, All India Institute of Medical Sciences, Raipur, Chhattisgarh, India; 21 Department of Community Medicine, Government Medical College, Surat, India; 22 Department of Community Medicine, IMHANS, Kozhikode, Kerala, India; 23 Department of Psychiatry, BMHRC, Bhopal, India; 24 Department of Community Medicine, Regional Institute of Medical Sciences, Imphal, Manipur, India; 25 Department of Community Medicine, Sawai Man Singh Medical College, Jaipur, Rajasthan, India; 26 Tamil Nadu Health Systems Project, Tamil Nadu, India; 27 Clinical Psychologist, M.S. Chellamuthu Trust, Tamil Nadu, India; 28 Department of Community Medicine, King George’s Medical University, Lucknow, Uttar Pradesh, India; 29 Department of Child Psychiatry, King George’s Medical University, Lucknow, Uttar Pradesh, India; 30 Department of Community Medicine, IPGME&R, Kolkata, West Bengal, India; George Institute for Global Health, INDIA

## Abstract

Understanding the burden and pattern of mental disorders as well as mapping the existing resources for delivery of mental health services in India, has been a felt need over decades. Recognizing this necessity, the Ministry of Health and Family Welfare, Government of India, commissioned the National Mental Health Survey (NMHS) in the year 2014–15. The NMHS aimed to estimate the prevalence and burden of mental health disorders in India and identify current treatment gaps, existing patterns of health-care seeking, service utilization patterns, along with an understanding of the impact and disability due to these disorders. This paper describes the design, steps and the methodology adopted for phase 1 of the NMHS conducted in India. The NMHS phase 1 covered a representative population of 39,532 from 12 states across 6 regions of India, namely, the states of Punjab and Uttar Pradesh (North); Tamil Nadu and Kerala (South); Jharkhand and West Bengal (East); Rajasthan and Gujarat (West); Madhya Pradesh and Chhattisgarh (Central) and Assam and Manipur (North East). The NMHS of India (2015–16) is a unique representative survey which adopted a uniform and standardized methodology which sought to overcome limitations of previous surveys. It employed a multi-stage, stratified, random cluster sampling technique, with random selection of clusters based on Probability Proportionate to Size. It was expected that the findings from the NMHS 2015–16 would reveal the burden of mental disorders, the magnitude of the treatment gap, existing challenges and prevailing barriers in the mental-health delivery systems in the country at a single point in time. It is hoped that the results of NMHS will provide the evidence to strengthen and implement mental health policies and programs in the near future and provide the rationale to enhance investment in mental health care in India. It is also hoped that the NMHS will provide a framework for conducting similar population based surveys on mental health and other public health problems in low and middle-income countries.

## Introduction

Robust and good quality data is an essential pre-requisite to plan, develop, implement, monitor, evaluate and strengthen mental health services globally and especially in Low- and Middle-Income countries (LMICs) like India. There is a strong need to understand the pattern of mental disorders prevailing, the consequent health-burden, as well as delineate gauge the currently available resources to plan and deliver services across the country. Alongside the existing National Mental Health Policy [1], the Mental Health Action Plan [2] and the recent promulgation of the Mental Health Care act 2017 [3] (replacing the earlier one) [4], along with increased budgetary allocation to mental health care in India, an understanding of mental health burden will pave the way to implement effective mental health services. Furthermore, with the expansion of state and district mental health programmes to all states and districts, there has been a felt need for good quality data to strengthen mental health services in India.

Earlier epidemiological surveys of mental disorders in India have methodological problems due to wide differences in study designs, sampling methods, instruments used, case definitions, cultural adaptations, data collection methods and statistical interpretations. In addition, most of these studies were regional or limited in their coverage. Reviews of previous epidemiological studies have highlighted the difficulty in arriving at precise national or state level estimates of mental health problems in the country [5–15]. The World Mental Health Survey, undertaken across 29 countries also included India; however, the published details of the study which sampled populations from only 11 sites across India are limited to a few centers[15, 16]. To this end, a large scale nationally representative study of the prevalence and characteristics of mental disorders in India, was imperative. In fact, the NMHS was initiated due to the impetus from policy makers, professionals and Parliamentarians of India to obtain reliable estimates of mental illness to accurately inform policies and legislation to drive upgradation of mental health resources and programmes. The Ministry of Health and Family Welfare, Government of India, commissioned the National Mental Health Survey (NMHS) during the year 2014–15 to be implemented by the National Institute of Mental Health and Neuro Sciences (NIMHANS), Bengaluru [17, 18]. The broad objectives of the NMHS were

- Estimating the prevalence and burden of mental disorders in a representative population of India
- Identifying the current treatment gap, existing patterns of health care seeking and service utilization patterns, along with an understanding of the impact and disability due to mental disorders in India, and
- Assessing mental health care resources and facilities in the surveyed Indian states for planning and strengthening mental health services in India

The NMHS was planned in three phases with the first phase being implemented in 12 representative states followed by surveys in the mega cities. It is planned to cover the remaining states in third phase. The second phase is proposed specially for the six metropolitan cities of India, namely New Delhi, Mumbai, Kolkata, Chennai, Hyderabad and Bengaluru.

The present paper explains the design, steps and the detailed methodology of the quantitative component of the first ever nation-wide mental health survey conducted in India during the period 2015–16. The detailed methodology of the qualitative component of the survey and mental health systems assessments along with the results of the survey will be part of our forthcoming publications.

## Methodology

The NMHS was undertaken on a representative population covering 12 states across 6 regions of India. The states included were from the Northern (Punjab and Uttar Pradesh), Southern (Tamil Nadu and Kerala), Eastern (Jharkhand and West Bengal), Western (Rajasthan and Gujarat), Central (Madhya Pradesh and Chhattisgarh) and North-Eastern regions (Assam and Manipur) of the country. The selected states comprised of diverse ethnicities varying in socioeconomic and cultural characteristics. The national survey included all individuals 18 years and above (and a limited sample of adolescents in 13–17 years in 4 states), in all the 12 selected states and used a combination of quantitative and qualitative methods to assess the burden of mental health problems and the status of mental health systems in India.

### Preparatory phase

#### Pilot study

A pilot study was conducted to assess the feasibility of the sampling design, appropriateness of intended survey instruments (Mini International Neuropsychiatric Interview (MINI) and Mini International Neuropsychiatric Interview for children and adolescents (MINI-KID) (and set of other data collection tools), training requirements for data collectors, data management issues, utility of employing electronic data collection on hand held tablet computers, and to obtain baseline estimates for calculating sample size and organise the logistics for the main survey [19]. It was conducted in Kolar district of the southern Indian state of Karnataka between February and December 2014. The pilot study revealed that systematic random sampling was more suited for household selection as in multi indicator cluster surveys [20] in comparison to other conventional nearest household selection method used in routine coverage surveys [21, 22]. It was also apparent that the MINI [23] and MINI-KID [23] could be used as one-stage screening and diagnostic instruments. Prevalence rates of 7.5% and a non-response rate of 30% for sample size calculation for the main survey and the need for systematic training of data collectors was ascertained from the pilot study. Based on the findings and experiences of pilot study, the NMHS Master Protocol and NMHS operational guidelines were developed with detailed specifications on the conduct of the NMHS in the different states.

#### Development of protocols and guidelines

The Master Protocol developed by the core team from NIMHANS was reviewed, finalised and ratified by the National Technical Advisory Group (NTAG) and the National Experts Panel [24]. The NTAG included domain experts from the fields of Psychiatry, Public health and Social Sciences and representatives of the Ministry of Health and Family Welfare. The NTAG provided technical inputs, direction and facilitated the implementation of the survey. In addition, a National Expert Panel consisting of biostatisticians, demographers and survey methodologists was constituted to examine, review and finalise the methodology, ensure quality and enabled finalizing the NMHS master protocol and operational guidelines.

To facilitate uniform implementation of Master Protocol for the national survey, an operational guidelines document was developed as a companion step-by-step guide to the NMHS master protocol as a manual on “how to do” the survey across the study sites [25]. The final version of NMHS Master Protocol was approved by the NIMHANS Institutional Ethics Review Board. In addition, each participating institution in the individual states obtained approvals from their respective Institutional Ethics Committee.

### Project management

**Fig 1:**
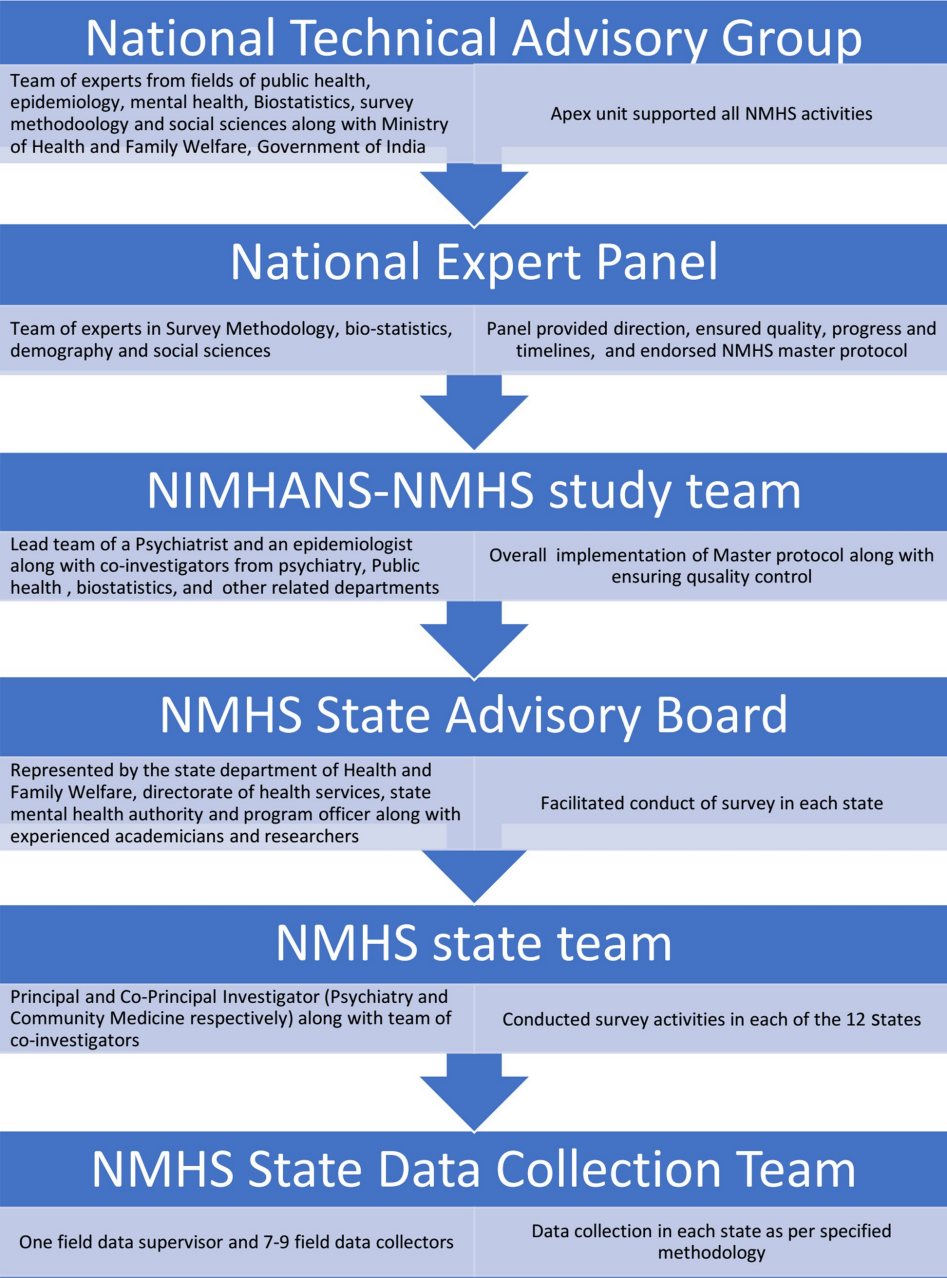
Project management–organogram with roles and responsibilities of different teams.

The overall project management organogram is provided in Fig 1. The NIMHANS-NMHS study team consisting of Principal Investigators (PIs) and Co-Principal Investigators (Co-PIs) was involved in overall preparation, coordination, management, implementation, support, monitoring, data management, analyses, and report development for the NMHS. In each state, the state mental health survey was led by the lead investigators drawn from the departments of Psychiatry and Community Health along with state mental health authority and administrative divisions. A state advisory board supported the administrative and operational aspects of the survey in each state. The state NMHS team employed and trained the state data collection team for data collection in each state.

Every component of the survey was discussed, reviewed for scientific rigour, feasibility in the field and approved by the NTAG, NEP, NAC, NIMHANS NMHS study team in consultation with the NMHS state teams.

### State study and data collection teams

The selection of states for Phase 1 of the NMHS was based on representation for different geographical regions of India (north, south, east, west, central and north-east), as well as the availability of interested partners to implement the survey as per protocols. Partners with previous experience in mental health research, knowledge of psychiatric epidemiology, field level experience in conducting large scale projects and programmes, willingness to participate and able to follow-up at state level in translating the research protocol to action were contacted and their participation solicited. In situations where there were two contiguous states with two competing teams, the state with the scarcer mental health morbidity data was preferred.

In each state, the lead Principal Investigator (PI) was a mental health professional and the co-principal investigator was a public health specialist. The PI and Co-PIs in each state jointly identified other co-investigators and co-opted other professionals / experts depending on local situations as per the directives of lead investigators. The NMHS state data collection team consisted of the NMHS state team and the NMHS field team (Fig 1). Overall, 104 field data collectors (FDCs) (8-member teams in 8 states undertaking adult surveys and 10-member teams in 4 states undertaking both adolescent and adult surveys) with a background in psychology (40%) / social work (50%)/ communications and rural development(10%) were recruited. Candidates with prior field data collection experience, ability to liaise with different stakeholders, and fluency in local languages and dialects were selected.

### Study instruments

The study instruments included:

1] A sociodemographic questionnaire developed to collect household and individual details based on the questions from the household questionnaire of the Census of India 2011 [26]. General household information collected included household number, cluster type (rural, urban and metro), period of residence in the current location, address, family composition, contact numbers of family members, income from all sources and usual source of treatment during illness. A unique ID was generated for each member in the household for further data capture. For each of the surveyed members, details of socio demographic information gathered included age in completed years, gender, education, occupation, income and marital status of the individual.
2] The MINI 6.0 for assessment of mental morbidity (including suicidal risk): Mental health morbidity was assessed using the MINI 6.0 [23, 27] for adult respondents (18+ years) and MINI Kid [27, 28] for adolescents (13–17 years). Both the MINI and MINI kid were selected following the experience of pilot study and recommendations by the NTAG and availability in local languages for the selected states. The MINI overcomes the impediment of two stage interviews needed in population based mental health epidemiological surveys [23, 28–30], provides ICD-10 –DCR [31] compatible diagnostic categories, takes lesser time and was found easy to administer following systematic training to the data collection team. Further, the MINI had the advantage of being available in multiple Indian language versions and had a digital version for administration on tablet computers.
3] The Fagerstrom questionnaires for tobacco use disorders-modified for recording both smoking and smokeless tobacco use[32]. The Fagerstrom questionnaire was chosen as it provides a measure of Tobacco Use Disorders, since the MINI does not enquire for tobacco use. The fact that it has been widely translated and used in the Indian setting, prompted its choice.
4] As epilepsy is routinely included in care delivery of mental health programmes, the WHO-SEARO screening questionnaire for Generalized Tonic Clonic seizures in community [33] was included as a screening instrument for epilepsy, to provide presumptive measures for epilepsy.
5) Similarly, a separate brief screening instrument developed by NIMHANS was utilized for intellectual disability (ID) and Autism Spectrum Disorder (ASD) screening, since there were no modules within the MINI. The screener questions of epilepsy, ID and ASD were used to obtain preliminary estimates of population prevalence of these conditions to aid future studies.
6) The Sheehan Disability Scale (SDS)- [34, 35] was used to measure levels of mental-health related functional impairment in primary care settings, developed to assess functional impairment in three inter-related domains; work/school, social and family life. The SDS has shown high internal consistency reliability and good construct validity [36]. It has been reported to have consistently high correlation with other scales such as the WHO Disability Assessment Scale [37]. Sheehan's Scale is resident on the MINI software platform with a built-in scoring algorithm, consistent with the rest of the MINI.
7) The instrument with regard to health care seeking and utilisation patterns was developed based on the experience of using the WHO-Pathways Interview Schedule (encounter form) [38] during the pilot survey. This instrument contained information on the duration of problems, current treatment provider, source of treatment, duration between onset and help seeking, number of treatment providers seen, details of current treatment provider and approximate expenditure for the treatment.
8) Section on socioeconomic impact of illness (modified based on WHO-DAS 2.0) [39] included a set of 7 questions looking at subjective reporting of overall difficulties, duration of these difficulties in the past 30 days, its impact on routine activities, expenditure due to illness, respondent missing on family, social or leisure activities due to illness.

All instruments were reviewed by the project team and state PIs in the beginning and also validated during the pilot study in Kolar [19]. Feasibility of application of MINI and other instruments by lay interviewers was validated against Psychiatrist evaluation during pilot survey. This was found to be in good agreement with psychiatrist evaluation [19]. The data collectors were trained using these instruments followed by a small pilot survey in all states prior to the survey to ensure local cultural and contextual appropriateness. A revalidation of the survey was done through re-interview of 5% interviews by psychiatrists (lead PIs at state level) in all the states.

#### Translation of study instruments

The interviews and data collection in each state was undertaken in local languages. Though translations of the MINI in some Indian languages were available, on reviewing them, the translations were found to be very general and at times failed in conveying the meaning of the questions to the general public. Hence, the vernacular versions had to be modified to suit survey requirements in state languages. Essentially, translation was required in seven Indian languages namely Assamese, Gujarati, Malayalam, Tamil, Punjabi, Bengali and Hindi. In five states, Hindi was the regional language and in Manipur, the English version of the instruments sufficed. The following steps were followed during the translation process.

1. The available official versions were reviewed by a team of experts at NIMHANS
2. The state team undertook a second review of the study instruments and made the necessary changes and of required modifications were identified
3. The required modifications were once again reviewed by the NIMHANS team and finalized during the national collaborators’ meeting through in-depth discussions
4. Field testing of the final version was undertaken in each state
5. This version was back translated using the standard WHO method and examined by the central team
6. These translated versions were finally pilot tested at each survey sites for fidelity
7. This approved version was incorporated into the handheld devices for data collection

Detailed information of the translation process is provided in the NMHS report [40]

### Sampling strategy

**Fig 2:**
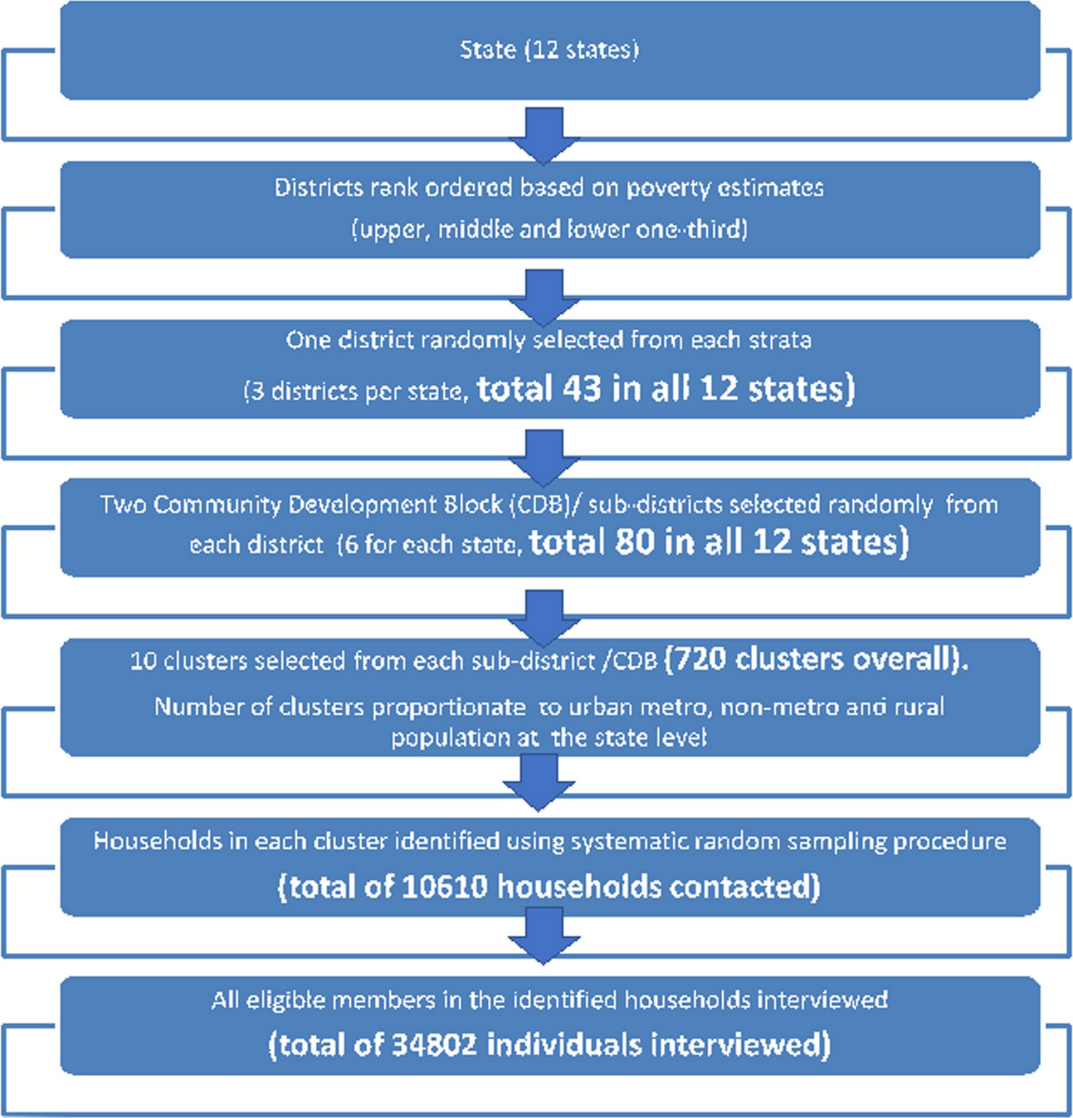
Overview of study designs.

A multi-stage, stratified, random cluster sampling technique, with random selection of clusters based on Probability Proportionate to Size was adopted to ensure representation of urban (metro and non-metro) and rural population in the sample (Fig 2).

Multistage sampling involved selection of districts, taluka/ sub-district (existing administrative divisions in Indian states), village/ ward and households with the household being the primary sampling unit. Stratification involved rural, urban-metro and urban non-metro clusters. Each named inhabited village as per the Census of India 2011 [41] constituted a rural cluster, while each urban ward from the Census 2011 constituted the urban cluster (metro & non-metro). Clusters in cities with million plus population as per census 2011 were considered as metro cluster, while those less than a million population was considered non-metro cluster [26]. The number of clusters chosen per stratum within a district was proportionate to the rural, urban-metro and urban non-metro population of the state. District stratification was done based on district level poverty estimates [42]. All districts within a state were ranked and divided into upper third, middle third and lower third and one district from each stratum was randomly selected. As a next step, from each district, two sub-districts were selected randomly (Fig 2).

### Sample size estimation

The sample size was computed using the prevalence of any mental health morbidity among adults of 7.5% (based on findings of the pilot study and earlier epidemiological surveys) with an absolute precision of 2% at 95% confidence and 30% non-response. The design effect was fixed at 3 for an assumed intra cluster correlation of 0.05. This design effect was assumed to account for clustering at the household level as well as the cluster level. The resulting sample size of 2857 was rounded off to 3000 for each state. Thus, 3000 adults above 18 years of age were targeted for data collection through direct interviews in each state. Overall, 34802 out of 39532 eligible individuals were interviewed in the identified households.

The NTAG recommended a pilot study of adolescents (13–17 years) including all adolescents in the same households selected for survey of adults in four states to obtain preliminary estimates on prevalence of mental health problems among adolescents. Thus, in total 36,000 adults and nearly 1200 adolescents drawn from 12 states were targeted for data collection.

### Households and respondent selection

The household was considered the primary sampling unit. A total of 60 clusters were identified and completed in each state with a target of 50 respondents per cluster to achieve the requisite sample of 3000. Households were selected using a systematic random sampling technique that was derived utilizing the household size as per Census 2011. All households within the cluster were first listed and then the sampling performed. In each selected household, the data collectors identified the head or any available responsible adult in the household. Line listing of all the members of the household along with collecting socio demographic information of all the members of the household was conducted. All eligible individuals were contacted for interview. After ascertaining the availability of all eligible members of the household, the interviewer initiated and completed the interview with all the individual eligible members of the household on all components of the survey after obtaining consent.After the first visit, the subsequent visits were made during holidays when the respondent was available or taking an appointment based on the time when the respondent was likely to be available. A maximum of three visits were made to interview a respondent, failing which he/she was declared a non-responder. All eligible respondents within the household were interviewed ensuring adequate privacy and confidentiality within the household or any place convenient to the respondent as appropriate to local situations. Efforts were made to ensure conduct of interview by the interviewer of the same gender as the respondent.

### The training process

Training for data collection was conducted at 3 levels (for the core team, state teams and for data collection teams) utilizing a specifically developed training manual. The training was conducted by a core team of epidemiologists and psychiatrists from NIMHANS experienced in conducting population-based surveys, use of MINI and other instruments, interview techniques as well as the use of tablet-based computers and in interviewing skills. The training for data collection team was undertaken over a period of 7 to 8 weeks (8 weeks in states where adolescent interviews were held) adopting adult learning principles using a uniform training schedule. The training schema included details of activities for each week with a provision to expand as required. The training conceptually relied on **SEE–PRACTICE–CONDUCT–REFINE** principle. FDCs **SAW** the process of interviewing for the first three weeks, **PRACTISED** conducting interviews from the end of the third week till the 5th week–**CONDUCTED** interviews independently under supervision in the 6^th^ and 7^th^ weeks and **REFINED** their skills in the 8^th^ week. Finally, FDCs were trained to interview patients and non-patients using the MINI and other instruments, both in hospital and community settings. The evaluation of training was done at three levels of -training related evaluation, objective assessment of the quality of interviews during the training and post-training evaluation by the resource persons team conducting the training in the field. Following training, each FDC was trained in general interviewing skills, understand survey procedures, administration of different survey instruments, obtaining consent and interview, documentation using hand-held devices and checking completeness of data collected. At the end, each FDC was certified for the satisfactory completion of the training and additional feedback/ training was provided whenever required.

### Data collection

**Fig 3:**
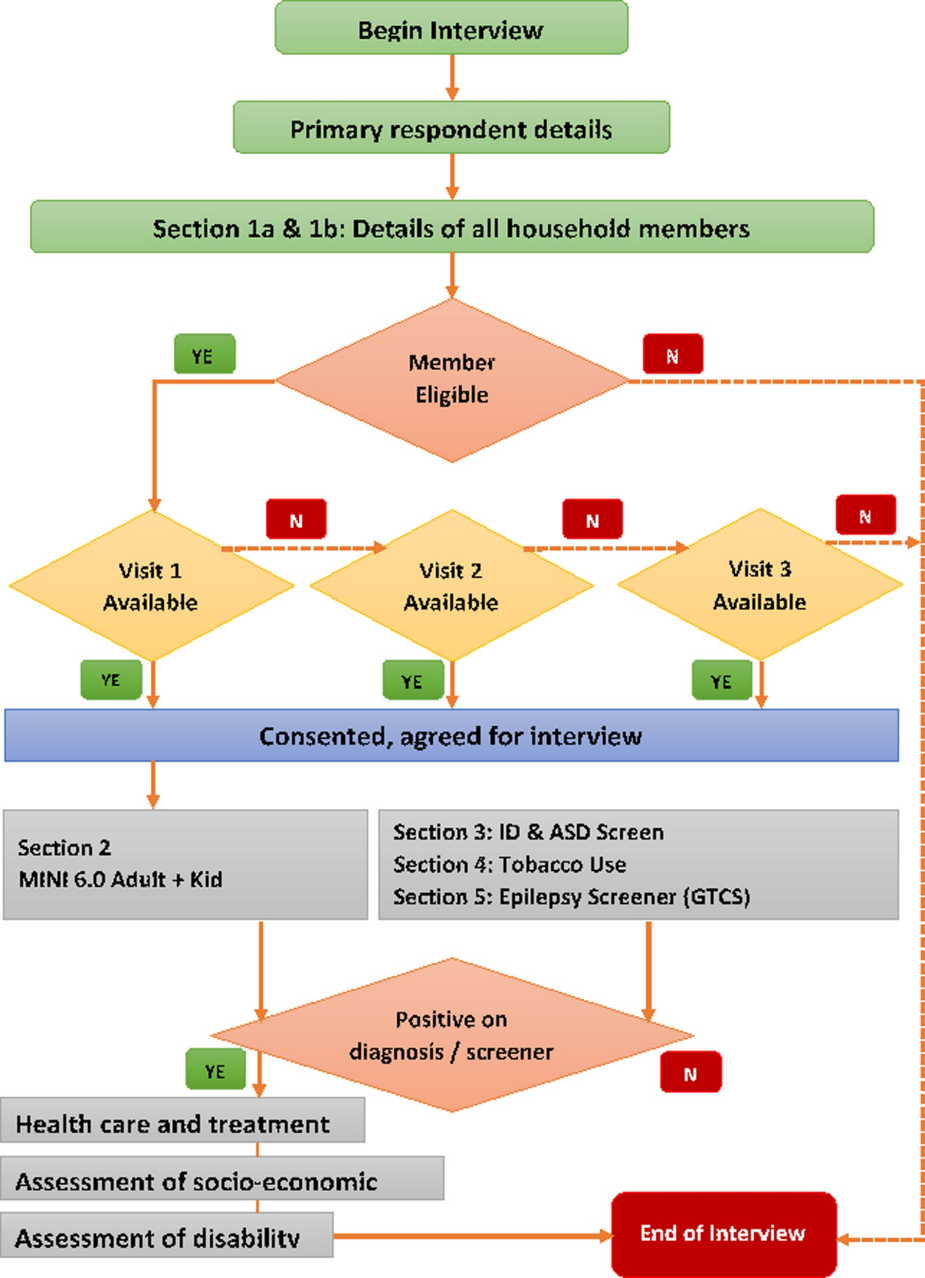
Steps in data collection and flow of interview.

The detailed steps of data collection followed in each state as per the sampling strategy is provided in Fig 3. A specific digital application was developed for data collection, real time entry and regular uploading of data. The algorithms for skips, NMHS criteria for morbidity and MINI diagnosis were in-built into this application (Fig 3). The FDCs, collected data using hand held digital computers eliminating the use of paper and pen, and allowing direct transfer to the central database thus avoiding errors due to manual data transfer. The household and individuals were contacted at least thrice, before declaring him/ her as non-respondent.

### Monitoring and quality assurance

A three-tier monitoring mechanism was adopted at the field, state and central levels to ensure quality of data collection. At the field level, spot checks were performed by the state teams as well as the field data supervisor. This included observation of interviews conducted by the field data collectors and review of their data monitoring forms. At the state level, the state investigators conducted re-interviews on 5% of all completed interviews along with weekly and monthly review meetings to address field level challenges and progress. The central team from NIMHANS conducted fortnightly review meetings with the state teams on an e-platform using video conferencing facilities to review progress of each state; appraise them about the quality of data collected and the receipt of uploaded data on to the NMHS server. A total of 218 online review meetings were conducted during the entire period of data collection by the central team at NIMHANS. The representatives from the NIMHANS NMHS team also visited the state and the field during data collection to ensure quality of data collection and adherence to the NMHS protocol.

### Data management

A separate and secure authenticated webpage was created with the domain name “indianmhs” (http://indianmhs.nimhans.ac.in) for the purpose of the survey with access limited to select responsible investigators from the NIMHANS NMHS team. The PI, Co-PI or the study coordinator transferred their respective state data onto the NIMHANS server at specified intervals that was monitored on a regular basis. Data received was checked for coverage, completeness, quality, errors, duplication and adequacy. Identified errors and duplicates were classified and corrected in consultation with the state teams. A separate log was maintained for all corrections and modifications to the data. This digital data management met ethical standards of human research and was approved by the ethics committee of NIMHANS as part of the protocol.

### Qualitative research

Instruments for quantitative component of the survey could not adequately capture granular information in a few vital areas like the regional nature and patterns of drug use and abuse, homeless mentally ill, stigma, health care utilization, under reporting and diverse cultural understanding and terminologies of mental illness in different areas. Thus, A qualitative component was included to capture information on these pre-identified domains to supplement the quantitative survey. The differing patterns of mental health care seeking among different communities and the widely divergent barriers/ challenges to seeking mental health care in different states were also examined. Key Informant Interviews (KII) and Focus-Group Discussions (FGD) were adopted for qualitative enquiry. A structured interview guide with a standard set of questions, probes, and lead points for both the KII and the FGD was developed for the qualitative survey. A separate manual on the conduct of qualitative survey with clear standard operative procedures for conduct of interviews and FGDs were also developed and field tested along with the master protocol for the survey [24].

In each state, 4–5 FGDs with mental health care providers, community members and others (in each district centre and 1 in state capital) drawn from both public and private institutions was completed. One KII (at the state capital) was conducted that included psychiatrists or specialists, pharmacists, state representatives, the police, legal and welfare sector, representatives from a local NGO and media. Both FGDs and KIIs were audio-recorded after informed consent of the participants. The findings in each state were summarized in a structured format and reported along with the photograph of the deliberations.

## Planned analysis

The International Classification of Disease, 10^th^revision, Diagnostic Criteria for Research (ICD-10 DCR) [31] was used to classify the different mental disorders in the NMHS. Current (Point) prevalence was reported for all diagnostic groups, while life-time prevalence (ever in the life of an individual in the past) for select conditions like bipolar disorders and psychotic disorders were reported as they were captured by MINI.

Any respondent was defined as having mental morbidity if s/he was found to be positive on one or more modules of the MINI for axis I disorders on ICD 10 –DCR criteria.

Further, the definitions adopted to classify different morbidities under NMHS are detailed in ANNEXURE A in S1 File.

As the primary objective of NMHS was to arrive at estimates of mental morbidity at both national and state levels, adjustment for non-responses inherent in such large surveys had to be factored. Thus, the sampling weight was estimated using the strategy provided in ANNEXURE B in S1 File. The National pooled estimates were calculated using the functionality of applying weights (weights on) in the Statistical Package for Social Sciences 18.0 (SPSS) [43] and re-checked with survey command in STATA 12.0 [44] statistical packages.

## Results

The NMHS was carried out during 2015–16 across 12 states which included 43 districts, 80 sub-districts, 729 clusters, 10152 households and 34802 individuals. Overall household response rate was 91.1% (range– 75.6% in the southern state of Kerala to 99.3% in the northern state of Punjab) with 10 states having more than 85% response rate (Table 1). The overall individual response rate was 88% ranging from 78.7% in Kerala to 96.1% in Rajasthan. Data collection was started on 2^nd^ October 2015 (after all preparations and planning in place) in western state of Gujarat and completed on 10^th^ June 2016 in the central state of Madhya Pradesh (Table 1).

**Table 1:** Sampling framework for the National Mental Health Survey– 2016.

	South		West		North		Central		East		Northeast		TOTAL
	KL*	TN*	GJ*	RJ*	PB*	UP*	CG*	MP*	JH*	WB*	AS*	MN*	
Number of Districts	14	32	26	33	20	71	18	50	24	19	27	9	343
Number of Districts Selected	3	4	3	4	4	4	3	4	4	4	3	3	43
Number of Taluka in the selected Districts	15	32	19	30	17	19	29	33	52	88	21	11	366
Number of Taluka Selected	6	7	7	7	7	7	6	7	7	7	6	6	80
Total number of Clusters in the Selected Taluka	265	1082	738	1200	1103	2544	1067	1239	967	966	1035	272	12,478
Number of Clusters selected	60	60	60	60	60	60	60	60	60	60	60	60	720
Proportion of clusters selected (%)	22.6	5.5	8.1	5.0	5.4	2.4	5.6	4.8	6.2	6.2	5.8	22.1	5.8
Number of Households in the Selected Clusters	192,569	76,322	360,678	49,184	76,161	68,033	50,603	62,462	58,281	89,017	34,594	51,971	1,169,875
Number of Households Contacted	1223	1083	953	602	723	880	738	1051	685	842	954	876	10610
Proportion of Households Contacted	0.6	1.4	0.3	1.2	0.9	1.3	1.5	1.7	1.2	0.9	2.8	1.7	0.9
Number of Households interviewed	926	1069	927	576	719	795	722	918	637	654	926	797	9666
Proportion of Households interviewed (%)	75.7	98.7	97.3	95.7	99.4	90.3	97.8	87.3	93	77.7	97.1	91	91.1
Number of eligible Individuals in the selected households (≥18 years)	3149	3462	3439	3233	3158	3788	3079	3240	3673	2818	3104	3389	39,532
Number of Eligible Individuals interviewed	2479	3059	3168	3108	2895	3508	2841	2621	3022	2646	2603	2852	34802
Proportion of Eligible Individuals interviewed (%)	78.7	88.4	92.1	96.1	91.7	92.6	92.3	80.9	82.3	93.9	83.8	84.2	88.0

*KL = Kerala; TN = Tamilnadu; GJ = Gujarat; RJ = Rajasthan; PB = Punjab; UP = Uttar Pradesh; CG = Chattisgarh; MP = Madhya Pradesh; JH = Jharkhand; WB = West Bengal; AS = Assam; MN = Manipur

The characteristics of the surveyed population are provided in Table 2. The overall sampling frame consisted of 343 districts from 12 states with 43 districts chosen randomly. Districts selected in five states had a metro city within the same districts while new districts with a metro city had to be selected in seven districts. Individuals aged 18–29 years formed the predominant age group in the survey. The sample proportion was similar to the national proportions (as per census 2011) [41] across all age groups, place of residence and literacy status. Nearly, 3/4^th^of the sample (74.7%) were currently married and 6.25% were widowed, separated or divorced.

**Table 2:** Socio demographic characteristics of study subjects selected for NMHS.

		Un-Weighted		Weighted		Un-Weighted		Weighted		Un-Weighted		Weighted	
Variables	Categories in Variable	Males		Males		Females		Females		Total		Total	
		n	%	n	%	n	%	N	%	n	%	n	%
Age group	18 to 29	5537	33.39%	215464	34.96%	6311	34.64%	241762	36.75%	11848	34.04%	457226	35.89%
	30 to 39	3377	20.36%	128809	20.90%	3685	20.23%	135483	20.60%	7062	20.29%	264292	20.74%
	40 to 49	2731	16.47%	101651	16.50%	3123	17.14%	110309	16.77%	5854	16.82%	211960	16.64%
	50 to 59	2088	12.59%	73847	11.98%	2360	12.95%	81418	12.38%	4448	12.78%	155265	12.19%
	60 and above	2852	17.20%	96479	15.66%	2738	15.03%	88867	13.51%	5590	16.06%	185346	14.55%
Place of Residence	Rural	11384	68.64%	404997	65.72%	12573	69.02%	437909	66.57%	23957	68.84%	842906	66.16%
	Urban non-metro	3162	19.07%	92483	15.01%	3439	18.88%	98215	14.93%	6601	18.97%	190698	14.97%
	Urban metro	2039	12.29%	118770	19.27%	2205	12.10%	121715	18.50%	4244	12.19%	240485	18.88%
Education	Illiterate	2450	14.77%	93486	15.17%	5959	32.71%	231236	35.15%	8409	24.16%	324722	25.49%
	Primary	3112	18.76%	131919	21.41%	3048	16.73%	121598	18.48%	6160	17.70%	253517	19.90%
	Secondary	3075	18.54%	112258	18.22%	2647	14.53%	93734	14.25%	5722	16.44%	205992	16.17%
	High School	3498	21.09%	114686	18.61%	2995	16.44%	89925	13.67%	6493	18.66%	204611	16.06%
	Pre-University	1916	11.55%	70107	11.38%	1598	8.77%	53622	8.15%	3514	10.10%	123729	9.71%
	Vocational	250	1.51%	10444	1.69%	109	0.60%	3668	0.56%	359	1.03%	14112	1.11%
	Graduate	1600	9.65%	59363	9.63%	1313	7.21%	46137	7.01%	2913	8.37%	105500	8.28%
	Post Graduate	450	2.71%	16954	2.75%	411	2.26%	14493	2.20%	861	2.47%	31447	2.47%
	Professional	188	1.13%	5317	0.86%	82	0.45%	1839	0.28%	270	0.78%	7156	0.56%
	Not known	46	0.28%	1716	0.28%	55	0.30%	1587	0.24%	101	0.29%	3303	0.26%
Occupation	Cultivator	2882	17.38%	109289	17.73%	376	2.06%	15255	2.32%	3258	9.36%	124544	9.78%
	Agricultural Labourer	2104	12.69%	79805	12.95%	927	5.09%	36665	5.57%	3031	8.71%	116470	9.14%
	Employer	327	1.97%	11493	1.86%	48	0.26%	1525	0.23%	375	1.08%	13018	1.02%
	Employee &other worker	6872	41.44%	245970	39.91%	3264	17.92%	77442	11.77%	10136	29.12%	323412	25.38%
	Student	1559	9.40%	60389	9.80%	1277	7.01%	49092	7.46%	2836	8.15%	109481	8.59%
	Household duties	227	1.37%	8108	1.32%	10227	56.14%	395091	60.06%	10454	30.04%	403199	31.65%
	Dependent	1210	7.30%	48183	7.82%	1548	8.50%	65148	9.90%	2758	7.92%	113331	8.90%
	Pensioner	649	3.91%	21475	3.48%	361	1.98%	10818	1.64%	1010	2.90%	32293	2.53%
	Others	755	4.55%	31538	5.12%	189	1.04%	6803	1.03%	944	2.71%	38341	3.01%
Marital status	Never Married	3903	23.53%	150698	24.45%	2614	14.35%	98334	14.95%	6517	18.73%	249032	19.55%
	Married	12235	73.77%	451105	73.20%	13745	75.45%	497750	75.66%	25980	74.65%	948855	74.47%
	Widowed/Divorced/ Separated	361	2.18%	11888	1.93%	1783	9.79%	59029	8.97%	2144	6.16%	70917	5.57%
	Others	86	0.52%	2559	0.42%	75	0.41%	2726	0.41%	161	0.46%	5285	0.41%
Total		16585	47.66%	616250	48.37%	18217	52.34%	657839	51.63%	34802	100.00%	1274089	100.00%

## Discussion

The National Mental Health Survey of India (2015–16) is a nation-wide representative survey conducted by adopting a uniform, standardized scientific methodology to arrive at estimates of mental morbidity and their related characteristics in India. The unique nature of the NMHS is its comprehensiveness, and that it provides vital information on the burden, treatment gap, health care seeking, service utilization patterns, disability status and impact of these disorders utilizing both quantitative and qualitative research methods. Furthermore, it also examined the preparedness and response to deliver mental health care to populations by examining mental health systems; all at one point of time.

The strength of the NMHS is that it overcame prevailing limitations of previous studies like small and varied sample sizes, limited populations, different time periods, different screening and diagnostic instruments, diverging statistical analyses and interpretations [8, 11, 12, 14, 30, 45]. Lately (year 2005 onwards), there have been attempts to conduct large scale surveys using validated instruments like the Composite international diagnostic interview schedule (CIDI), Structured clinical interview for DSM-IV axis 1 disorders (SCID-1) and General health questionnaire in few select countries [46–53]. Globally, the sample size varied from 2857 in Lebanon [49] to 63,000 in China [52]. NMHS is by far the largest in India and the second largest mental health survey undertaken in terms of sample size. This was accomplished by undertaking a pilot study, determining adequate sample size, scientifically determined sampling methods, inclusion of urban-metro-rural populations, utilizing valid and uniform study instruments that were translated into local languages, adopting standardized procedures for training and data collection across all study sites at one specified time period, thus ensuring representativeness, uniformity and standardization in a large and diverse country like India. Furthermore, the NMHS was conducted using a sampling strategy that was representative (12 states), stratified (3 districts in each state based on poverty index), random (2 Community Development Blocks / Sub-districts in each district and 10 clusters in each), proportional (rural, urban-metro & urban non-metro) including all individuals above 18 years (13+ years in 4 states).The sampling distribution was similar to the population distribution of census of India 2011[41]. Furthermore, the socio-demographic characteristics of those surveyed and not surveyed were observed to be similar.

Implementing a large scale nation-wide survey required a strong coordination and networking of professionals and administrators for implementing several activities in a timely manner. NMHS established a robust mechanism to develop, guide, supervise and coordinate all its activities. Multi-disciplinary teams with the right mix of experience and expertise were identified at different levels and brought together to achieve the stated objectives of the NMHS (Fig 1). In parallel, the policy makers were also part of the conceptualization, planning, process and progress of survey that is extremely essential for translating research to actionable programs.

In India, there has been a shift from small scale surveys to large scale surveys, comprehensively looking at problems or diseases of public health importance [54–56] in recent times. However, there was no such survey done for mental health problems in India, except the World Mental Health Survey undertaken 10 years ago. The NMHS is an attempt to bridge this gap and to look at epidemiological characteristics and patterns almost 10 years later. NMHS moved beyond prevalence estimates to also identify the current treatment gap, health care seeking and service utilization patterns, along with an assessment of mental health systems in surveyed states of India. The survey comprehensively examined almost all mental health problems of public health importance (including substance use disorders). Epilepsy was included as part of the survey since, epilepsy has traditionally been part of service delivery in the National Mental Health Program as well as recommended under the WHO mhGAP programme [57]. Additionally, the NMHS also focused on delineating service utilization patterns, disability status, the impact of mental disorders on individuals and families and the prevailing stigma in society. The focus on inclusion of assessment of current status of delivery of mental health services and systems with a focus on requisite human, financial, physical and other resources was felt essential by policy makers and programme managers for mental health service delivery.

Previous mental health surveys on prevalence of mental disorders in India have used variety of case detection tools (from unstructured to highly structured ones), each with its strength and limitations as well as a 2-step methodology of screening (by different categories of data collection teams) and evaluation (through different methods ranging from interviews to structured diagnostic tools). The NMHS used a standardized version of the MINI as well as additional instruments in a uniform manner in all surveyed states. The MINI is a structured diagnostic interview instrument for screening and diagnosing mental disorders both as per the DSM IV TR [58] and ICD– 10[31] and available on a digitised platform in different Indian languages, which required adaptation in a systematic manner. In the past, the MINI has been used in population based mental health surveys and has an acceptable level of clinometric properties [59, 60]. The MINI adult version was found suitable for the community based epidemiological survey as against MINIplus[23] which is more suited for in-depth clinical interviews [16, 29, 61, 62]. The MINI has separate versions for interviewing adults and children less than 18 years of age (MINI Kid). The CIDI instrument used in World Mental Health Survey was deemed to be too lengthy by the PIs and NTAG members. Furthermore, as the MINI uses an algorithm to provide the diagnosis, it was considered appropriate for utilisation under National Mental Health Survey. This was also tested for applicability in the field during the pilot survey in Kolar [19].

The NMHS utilized digital technology for the survey by using hand-held tablets for data collection and adapting online transfer of data from different locations. The digital hand-held tablets used for data collection reduced use of paper, saved time on data entry and reduced errors during data collection and entry with all the skip logics in place. Digital devices ensured speed, facilitated online data transmission, and helped in providing regular feedback for data collection teams. The pilot survey revealed that use of digital devices are also cost effective for eliminating the use of paper version in diverse field conditions. The fortnightly e-meetings with the state teams ensured discussion on progress, monitor and troubleshoot issues related to the survey.

The NMHS had certain barriers and challenges as well. Major challenges included overcoming prevailing socio-political tensions in certain clusters/ districts which made the survey difficult (two districts and six clusters were replaced after consultation with the district authorities and the PIs of the state), visiting villages and households that were interior (good planning helped), interviewing identified respondents (overcome through provision of information and repeat visits), ensuring privacy during interviews and working in adverse field situations.

This survey is not without limitations. (i) Firstly, including every state of the country would have been ideal to obtain precise estimates as states in India are formed considering linguistic and geographic boundaries. However, the selected 12 states in phase 1 were representative of different regions in the country and the remaining will be covered in the next phase of NMHS. The national estimates from these 12 surveyed states are likely to mirror the burden in the remaining states. (ii) The large cities of India with population of more than 10 million (metropolises) were not included in this survey since it was recognized that it requires different methodology for larger urban population, given the complex nature of urbanization and its effect on mental health.(iii) Children less than 13 years were not included in NMHS due to lack of clear understanding of mental disorders from a population perspective; absence of suitable and culture specific instruments; and lack of experienced teams to investigate child mental health issues. However, a pilot study of adolescents aged 13–18 years was conducted in 4 states of India to aid development of appropriate methodologies for future studies. (iv) In terms of the instruments used for the survey, the Fragerstrom Nicotine dependence scale is known to underestimate milder forms of tobacco use. However, the fact that it has been widely translated and used in the Indian setting, prompted its choice. (v) the MINI and MINI KID were not validated in populations where survey was undertaken. However, in overall terms, it was examined through the pilot study, at the beginning and end of training process, prior to commencement of survey in the community and during 5% re- interviews. (vi) Finally, this survey did not include the homeless mentally ill and institutionalized populations. However, recognizing its importance, a qualitative component was included in the NMHS.

In conclusion, the scientific, uniform and standardized methodology adopted by NMHS of India 2015–16 will reveal the burden of mental disorders, gaps, challenges and barriers in health seeking for mental health problems along with a status assessment of mental health systems in the country at the same time. This data will serve as evidence to strengthen and implement mental health policies and programs for the coming years as well as enhance investment in mental health care in India. NMHS also provides a framework for conducting similar population based mental health surveys and other public health problems in many low and middle-income countries that face a disproportionate burden in their populations.

## Supporting information

S1 File Supporting information file containing two ANNEXURES: ANNEXURE A–Case definitions used for different morbidities under NMHS ANNEXURE B: Sampling weight estimation.
(DOCX)
