# Supplementary material for: National Mental Health Survey of India, 2016 - Rationale, design and methods
Source: PLoS One. 2018 Oct 25;13(10):e0205096. doi: 10.1371/journal.pone.0205096 (PMC6201882; doi:10.1371/journal.pone.0205096)
Supplement: S1 File — Supporting information file containing two ANNEXURES: ANNEXURE A–Case definitions used for different morbidities under NMHS ANNEXURE B: Sampling weight estimation. (DOCX) [file pone.0205096.s001.docx]

ANNEXURE A – Case definitions used for different morbidities under NMHS

1. Any mental morbidity was defined as those disorders as per ICD 10 classification and captured by MINI instrument across 17 modules for axis I disorders. This represents the mental health morbidity including substance use disorders excluding tobacco use.
2. Common mental disorders include depressive disorders (mild, moderate and severe without psychotic features), Neurotic and stress related disorders and alcohol and other substance use disorders.
3. Severe mental disorders include Schizophrenia and other psychotic disorders, bipolar affective disorders and severe depression with psychotic features.
4. Comorbid mental morbidity was defined as presence of combined diagnosis on more than one mental health condition as identified by MINI instrument
5. Any Substance use disorders include alcohol use disorders, other substance use disorders and tobacco use disorders. Further,
   1. Alcohol use disorders include both dependence and harmful use
   2. Any substance use disorder includes both dependence and harmful use for other substances and includes the broad categories of opioids, cannabinoids, inhalants and prescription drugs
6. Substance abuse included tobacco use, alcohol abuse and dependence and the use of all illicit and prescription drugs.
7. Substance dependence was defined as any person who uses any substance and has three or more of the following characteristics for a particular substance within the last 12 months
   1. Tolerance (marked increase in amount; marked decrease in effect)
   2. Characteristic withdrawal symptoms; substance taken to relieve withdrawal
   3. Substance taken in larger amount and for longer period than intended
   4. Persistent desire or repeated unsuccessful attempt to quit
   5. Much time/activity to obtain, use, recover
   6. Important social, occupational, or recreational activities given up or reduced
   7. Use continues despite knowledge of adverse consequences (e.g., failure to fulfil role obligation, use when physically hazardous)
8. Suicidal risk was categorised as low, moderate and severe risk based on the scoring obtained in the MINI schedule. Low risk suicidality was defined as a score between 1 and 8 for the MINI schedule, while moderate and high risk of suicidality was defined as scores between 9 and 16 and more than or equal to 17, respectively.
9. In addition to defining operational definition of several disorders, the prevalence was classified based on the presence of disorders as per current time-period (point prevalence) or any time in the life of an individual in the past (life time prevalence).

ANNEXURE B: Sampling weight estimation

The sample size calculation permitted state level estimates of select mental health morbidity and pooled estimates at the national level. The sampling estimates are calculated utilising district and taluka selection probability using the formula

*NPW*=(*D_wi_ *IRR*_w_)

Where,

*NPW* is the National Pooled weight

*D_wi_*is the Design weight, calculated using the formula

D*_wi_* = 1/ (P_di_* P_ti_)

Where P_di_ is the probability of selection of a district within each strata of the poverty index calculated using the formula

*P_di_=n_di_/N_d_*

Where,

‘n_di_’ is the number of districts selected from ‘i_th_ ’division;

‘N_d_’ is the total number of districts in the ‘i_th_’division.

and P_ti_ is the probability of selection of a taluka within the selected district calculated using the formula

*P_ti_=n_ti_/N_t_*

Where,

‘n_ti_’ is the number of sub-districts selected from ‘i_th_’ selected district;

‘N_t_’ is the total number of sub-districts in the ‘i_th_’district.

*IRR_w_*is the weight for the individual response rate, calculated using the formula

IRR_w_ = 1/IRR

Where, IRR is the individual response rate.
